# A Model Study on the Possible Effects of an External Electrical Field on Enzymes Having Dinuclear Iron Cluster [2Fe-2S]

**DOI:** 10.1100/2012/985958

**Published:** 2012-05-01

**Authors:** Lemi Türker

**Affiliations:** Department of Chemistry, Middle East Technical University, 06531 Ankara, Turkey

## Abstract

Hydrogenases which catalyze the H_2_ *↔* 2H^+^ + 2e^−^ reaction are metalloenzymes that can be divided into two classes, the NiFe and Fe enzymes, on the basis of their metal content. Iron-sulfur clusters [2Fe-2S] and [4Fe-4S] are common in ironhydrogenases. In the present model study, [2Fe-2S] cluster has been considered to visualize the effect of external electric field on various quantum chemical properties of it. In the model, all the cysteinyl residues are in the amide form. The PM3 type semiempirical calculations have been performed for the geometry optimization of the model structure in the absence and presence of the external field. Then, single point DFT calculations (B3LYP/6-31+G(d)) have been carried out. Depending on the direction of the field, the chemical reactivity of the model enzyme varies which suggests that an external electric field could, under proper conditions, improve the enzymatic hydrogen production.

## 1. Introduction

Electron-transfer reactions are vital to many of the metabolic processes which are indispensable for the survival of organisms. These reactions depend upon the behavior of an electron acceptor and donor and are classified into two types (i) inner-sphere electron transfer in which the coordination spheres of the reactants share a ligand transitorily and so form a bridge intermediate; (ii) outer-sphere electron transfer in which the coordination spheres of the reactants remain intact [1]. The later process is most often found in biology, and biological electron transfers have been grouped in two categories; (i) intramolecular electron transfer which occurs at fixed sites within a single protein; (ii) intermolecular electron transfer which occurs between sites on different proteins [1]. The second process mentioned involves electron-transfer chains which operates via consecutive electron-transfer reactions between pairs of various proteins. Typical ones involve electron transfers between metal sites that are arranged within a protein or complex of protein.

**Figure 1:**
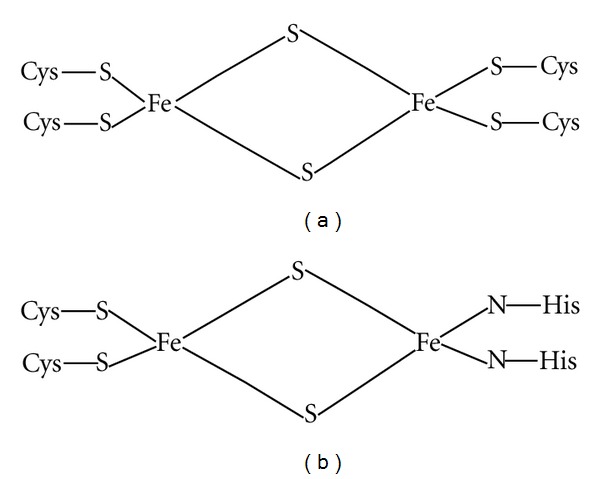
Some systems having [2Fe-2S] core.

Iron-sulfur proteins are involved in many electron transfer processes occurring in all living organisms [2–13]. They are nonheme proteins. They participate in various important oxidation-reduction processes such as nitrogen fixation and electron transfer in mitochondria. The iron atom present in these proteins is bound by sulfur atoms either from cysteinyl residues present in the structure of the protein moiety or by inorganic sulphides (see Figure 1(a)). One of subclasses of iron sulfur proteins is [2Fe-2S] dinuclear iron type [2–11], and those have been isolated from mammalian, plant, and bacterial sources and possess a common edge provided by bridging inorganic sulfur atoms. The remaining coordination sites are occupied by cysteinyl residues. The cluster transfers and accepts only one electron to produce [Fe (II)-Fe (III)] cluster [1]. Another subclass of [2Fe-2S] proteins has been isolated from bovine mitochondria and in their structures one iron atom linked to the cysteinyl residues and the second iron linked to two histidine residues (see Figure 1(b)) [1, 3, 6].

The [2Fe-2S] type clusters have been the focus of many theoretical studies [14–19]. Many of them are based on some model structures having suitable ligands attached to Fe centers. Using broken-symmetry DFT method, it has been found that structural variations of the ferrous center may dramatically affect the oxidation energy of the [2Fe-2S] clusters [14].

In the present study, a model structure for the [2Fe-2S] cluster has been considered to visualize the effect of external electric field on the various quantum chemical properties of it. Thus, one might get some idea about the behavior of such a cluster having biological systems in a permanent or transient external electrical field.

## 2. Method

In the present treatise, the geometry optimizations of all the structures leading to energy minima were achieved by using first molecular mechanics (MM+) and then PM3 [20] treatment. In the model, all the cysteinyl residues are in the amide form. The optimizations were achieved by the application of the steepest-descent method followed by conjugate gradient methods, Fletcher-Reeves and Polak-Ribiere, consecutively (convergence limit of 4.18 × 10^−5^ kJ/mol. (1 × 10^−5^ Kcal/mol.) and RMS gradient of 4.18 × 10^7^ kJ/M.mol. (1 × 10^−3^ Kcal/A.mol.), for each set of calculations, vibrational analyses were done. The normal mode analysis for each structure yielded no imaginary frequencies for the 3*N*−6 vibrational degrees of freedom, where *N* is the number of atoms in the system. This indicates that the structure of each case corresponds to at least a local minimum on the potential energy surface.

The geometry-optimized model structure was oriented in the *X* (horizontal direction) having an imposed restrain on the Fe-Fe direction and distance (2.673 Å, which is the Fe-Fe distance in the geometry-optimized model in the absence of any external electrical field), and then it was subjected to an electrical field (0.001 au) separately in the *X*, *Y* (vertical), and *Z* (vertical to the screen surface) directions. The model was reoptimized in the field using PM3 method. This treatment was repeated separately for *X*, *Y*, and *Z* field directions, and the optimized geometries were recorded. In the next step, these structures were subjected to single point B3LYP/6-31+G(d) type calculations. All the geometry optimizations in the absence or presence of the field were done by using Hyperchem 7.5 package program [21], whereas all the single point DFT calculations were performed by using Spartan 06 package program [22].

## 3. Results and Discussion

**Figure 2:**
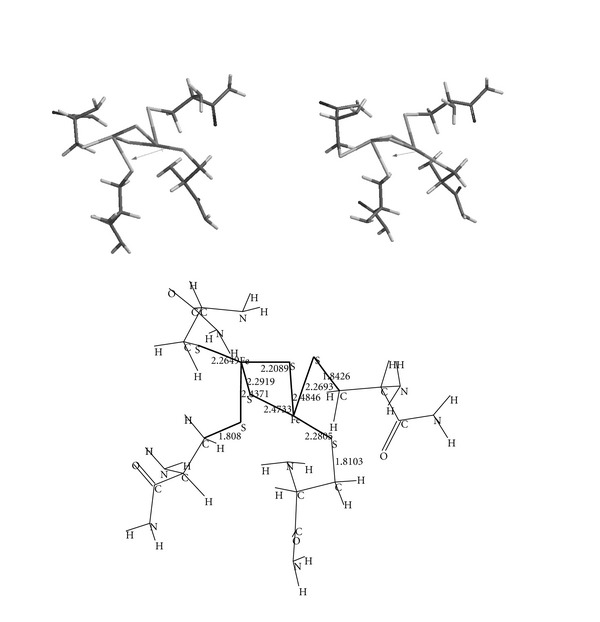
The geometry-optimized structure of the model in the absence of any external electrical field.

Valuable contribution to the understanding of the structure and reactivity of the enzymatic center comes from the model compound studies. In the present study, the model compound contains cysteinyl residues in the amide form.  Figure 2 shows the structure of the model in the absence of any external field. 

The figure displays also some selected bond lengths (in $\overset{´}{\text{Å}}$) and the direction of the dipole moment (6.65 D). Note that Fe-Fe distance is 2.673 $\overset{´}{\text{Å,}}$ whereas S-S distance of [2Fe-2S] core is 3.809 $\overset{´}{\text{Å}}$ in the geometry-optimized unperturbed system (no external field). It is also noteworthy to mention that although the substituents are constitutionally identical, the optimized geometry belongs to C1 molecular point group only. The optimized geometries in the external field also exhibit C1 symmetry. 

**Figure 3:**
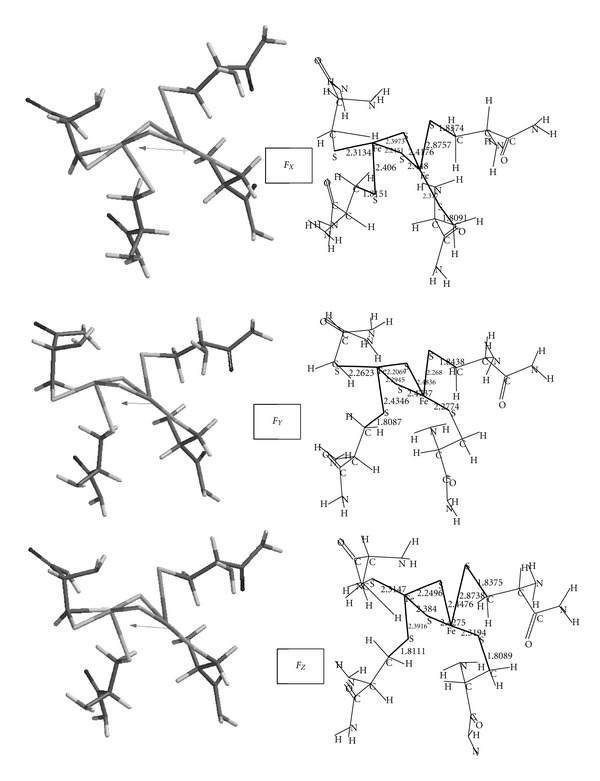
The geometry-optimized structure of the model in the presence of external electrical field applied along the coordinate axes. The arrows stand for the dipole moments. Some distances and bond lengths are also shown.

Figure 3 shows the geometry-optimized structures in the presence of external electrical field applied on the *X*, *Y*, and *Z* directions (indicated as *F*_*X*_, *F*_*Y*_, and *F*_*Z*_ in the figure). Also, some selected bond lengths are shown in the figure. Note that the external field presently was set to 10^−3^ au to prevent any drastic change in the geometry; thus, the geometry optimization in the field was facilitated somewhat.  Table 1 shows Fe-Fe and S-S distances in the [2Fe-2S] core of the model. As seen there when the field direction was along the *Y* axis, the above-mentioned distances were perturbed insignificantly as compared to the distances of the model exposed to electrical field in the *X* and *Z* directions. 

Table 2 shows the energies of the model in the absence of any electrical field and the applied field along the coordinate axes (B3LYP/6-31+G(d) single point calculations based on PM3 geometry optimization). As seen there, application of the field along the *X* and *Z* axes causes a little bit stabilization of the structures. The effect should be the combined effect of dipole-field interaction and reorganization of the geometry and molecular field in the external electrical field (*E*). However, as seen in Figure 3 and Table 3, *Y* component of the dipole moment is smaller than *X* and *Z* components in the unperturbed model. Therefore, the interaction energy (*ε*) of the dipole moment (*μ*) with the electrical field (*ε* = −*μE* cos⁡*θ*) is initially expected to be less in absolute magnitude. In the external field, the total dipole moment in every case is less than the respective value of the unperturbed model. 

Table 4 shows the HOMO and LUMO energies of the model. Application of the electrical field along the *X* direction lowers the HOMO but raises the LUMO energy level as compared to the unperturbed model. The effect of the electrical field along the *Y* direction is negligible on both the HOMO and LUMO energies, whereas the field along the *Z* direction lowers the HOMO and raises the LUMO as it was the case along the *X* direction. Application of the external field polarizes the bonds thus changes the electron densities. Consequently, energies of the molecular orbitals are affected as well as their shapes.

**Figure 4:**
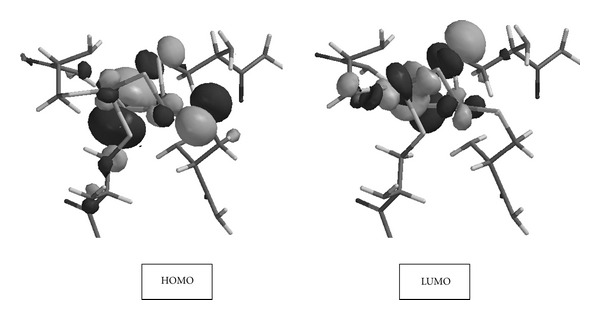
The HOMO and LUMO of the model in the absence of any external electrical field.

**Figure 5:**
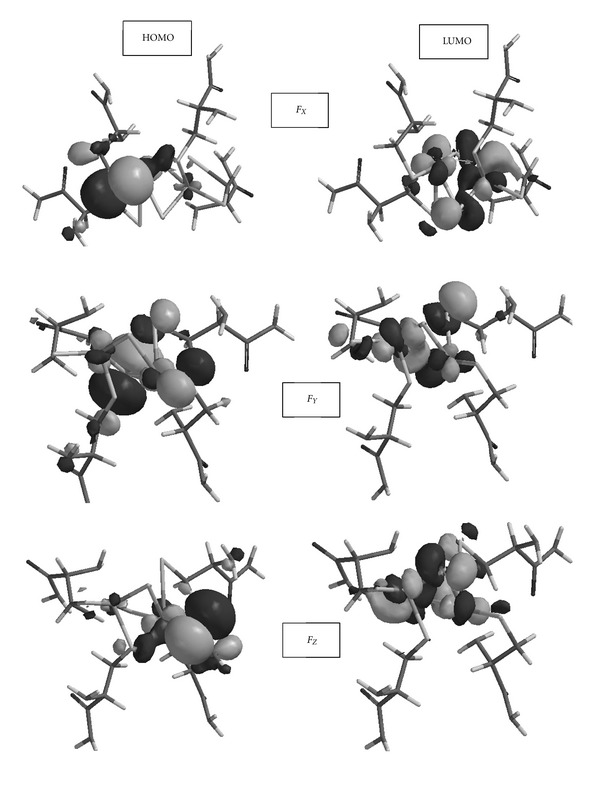
The HOMO and LUMO in the presence of external electrical field applied along the coordinate axes.

Figure 4 shows the HOMO and LUMO of the unperturbed model. As seen there, both of these orbitals possess contributions from atoms around the [2Fe-2S] core.  Figure 5 shows the HOMO and LUMO of the model exposed to external electric field along the coordinate axes. The HOMOs in the cases of *F*_*X*_ and *F*_*Z*_ are distinct from the HOMO of *F*_*Y*_ case, whereas the LUMOs display similar appearance. The main feature of these LUMOs is that they arise from the atomic contributions around the [2Fe-2S] core.

Table 4 also shows the interfrontier molecular orbital energy gaps (Δ*ε*) and the molecular hardness. The absolute hardness of a species is defined as

(1) $$\eta_{\circ }=\frac{1}{2}{\left(\frac{\partial \mu }{\partial N}\right)}_{v}=\frac{1}{2}{\left(\frac{\partial^{2}E}{\partial N^{2}}\right)}_{v},$$

where *μ*, *E*, *v*, and *N* are the chemical potential, energy, potential, and the number of electrons, respectively [23]. The finite-difference approximation for hardness is expresses as [20]

(2) $$\eta =\frac{I-A}{2},$$

where *I* and *A* are the ionization potential and electron affinity, respectively. The intramolecular hardness (*η*) or simply molecular hardness, involving one molecule has been widely used in the literature as a reactivity index in various types of systems (e.g., molecular vibrations, aromaticity, etc.) [24–30]. It has been shown that *η* predicts the reactivity of a system leading also to principles of maximum stability as it occurs with the maximum hardness principle [31]. The definition of molecular hardness in terms of energy of the frontier molecular orbitals is [23]

(3) $$\eta =\frac{1}{2}\left(\varepsilon_{\text{LUMO}}-\varepsilon_{\text{HOMO}}\right).$$

**Figure 6:**
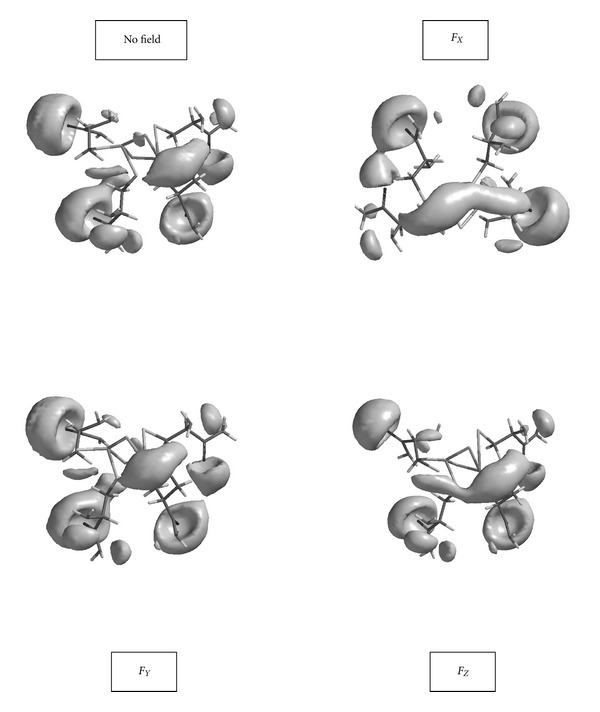
Potential field surface of the model in the absence and presence of the external electric field.

As seen in Table 4, *η* values follow the order of *F*_0_<*F*_*Y*_<*F*_*Z*_<*F*_*X*_. Thus, the softest species is the model in the absence of any external field and the hardest one is the system in the external field oriented in the *X* direction. Note that, presently, *X* axis is the primary axis passing through the center of mass of the molecular system. In general, the primary inertial axis is the longest distance from the center of mass to the edge of a molecular system [32]. Since the model studies predict that the system indicated by *F*_*X*_ is the hardest of all, then the hard-hard interactions will grow when the applied external field is in the *X* direction. On the contrary, in the absence of any external field, soft-soft interactions could be more likely if the overall interaction is not predominantly charge controlled.  Figure 6 shows potential field surface of the model in the absence and presence of the external electric field.

 The B3LYP/6-31+G(d)//PM3 type single-point calculations for the orbital energy of proton in the absence and presence of an external electric field having the same magnitude as the field used for the present model (0.001 au) yield −13.55761 eV and −13.55196 eV, respectively.

 Klopman-classified electron-transfer reactions as “charge-controlled” and “frontier orbital-controlled.” The first type reaction is characterized by a large difference between the frontier molecular orbitals of the donor and acceptor molecules. The second type occurs when the frontier orbitals of donor and acceptor molecules are close in energy [33].  Table 5 shows the Δ*ε* (*ε*_HUMO/model_ − *ε*_LOMO/H^+^_) values for the model-proton interaction in the absence and presence of the field. To visualize the influence of the applied external electric field, the effect of the enzyme model on H_2_ *↔* 2H^+^ +2e^−^ reaction has been considered.

 As seen in the table, the application of the external field turns proton to be somewhat softer, whereas the models described by *F*_*X*_ and *F*_*Z*_ get harder as compared to *F*_0_. The net effect is narrowing of Δ*ε* for donor-acceptor interaction. Thus, the interaction of the model and proton acquires some orbital control in the order of *F*_*X*_>*F*_*Z*_>*F*_*Y*_>*F*_0_, although the major type of interaction still could be charge controlled. All these calculations suggest that application of an external electric field may influence the H^+^ + e *→* H reduction, by increasing the contribution of the orbital control term.

Table 6 shows the *ε*_LUMO/model_ − *ε*_HOMO/H_2__ values for the oxidation of H_2_ molecule by the model in the absence and presence of the external field. For the geometry optimization of H_2_ molecule, the same calculation protocol as the geometry optimization of the model structure was applied (B3LYP/6-31+G(d)//PM3). The field applied (0.001 au) did not cause any significant change on the frontier orbital energies H_2_ molecule. The LUMO energy raised up very little. Δ*ε* values (*ε*_LUMO/model_ − *ε*_HOMO/H_2__) are in the order of *F*_0_ < *F*_*Y*_ < *F*_*Z*_ < *F*_*X*_. Hence, the application of the field decreases the orbital control in the oxidation of H_2_ molecule to proton. Therefore, the presence of an external field generally results in favorable proton reduction rather than the favorable oxidation of H_2_.

## 4. Conclusion

The present model study for the enzymes having dinuclear iron cluster, [2Fe-2S], has put some light on the effect of an external electric field on the molecular orbital properties. As compared to genuine enzyme systems, the model presently used is a simple one. Additionally, the [2Fe-2S] core is usually buried in the bulk of the bundle of proteineous chains, which might exert some sort of screening effect to the field applied. Therefore, the field strength presently used is comparatively held low (0.001 au). However, the applied field may polarize the suitable sites to generate local field effects whose directions and magnitudes cannot be predicted in real systems. Thus, although the effect of external field presently goes parallel with the favored proton reduction to produce hydrogen, if the enzyme particularly is responsible for that reaction, experimentation is required to visualize the magnitude on specific enzyme systems.

## Figures and Tables

**Table 1:** Some distances in the model enzyme in the absence and presence of external electrical field (B3LYP/6-31+G(d))//PM3.

	Fe-Fe	S-S
No field (*F*_0_)	2.673	3.809
*F*_*X*_	2.564	3.742
*F*_*Y*_	2.677	3.808
*F*_*Z*_	2.563	3.743

**Table 2:** The total energy of the model in the absence and presence of the external electrical field (B3LYP/6-31+G(d) single point calculations).

	No field (*F*_0_)	*F*_*X*_	*F*_*Y*_	*F*_*Z*_
Energy (au)	−6129.39159	−6129.44639	−6129.39278	−6129.44378

**Table 3:** Effect of external electrical field on the dipole moment (B3LYP/6-31+G(d) calc.).

	Dipole moment components (Debye)			Total (Debye)
	*X*	*Y*	*Z*	
No field (*F*_0_)	6.164784	−1.443091	2.048133	6.654731
*F*_*X*_	4.073490	1.965731	−0.672029	4.572665
*F*_*Y*_	5.803073	−1.662552	2.685401	6.606899
*F*_*Z*_	4.608336	−2.570551	−0.217747	5.281279

**Table 4:** The HOMO and LUMO energies, interfrontier energy gaps (Δ*ε*), and molecular hardness (*η*) of the model (B3LYP/6-31+G(d)).

	HOMO (eV)	LUMO (eV)	Δ*ε* (eV)	*η* (eV)
No field (*F*_0_)	−5.54013358	−3.99394885	1.5462	0.7731
*F*_*X*_	−5.61604421	−3.73847561	1.8776	0.9388
*F*_*Y*_	−5.54044079	−3.99268815	1.5478	0.7739
*F*_*Z*_	−5.58665173	−3.73864011	1.8480	0.9240

Δ*ε* = *ε*_LUMO_ − *ε*_HOMO_, *η* = 0.5Δ*ε*.

**Table 5:** The *ε*_HOMO/model_ − *ε*_LUMO/H^+^_ values (in eV) in the absence and presence of the external field.

No field (*F*_0_)	*F*_*X*_	*F*_*Y*_	*F*_*Z*_
8.0174	7.9359	8.0115	7.9653

**Table 6:** The *ε*_LUMO/model_ − *ε*_HOMO/H_2__ values (in eV) in the absence and presence of the external field.

No field (*F*_0_)	*F*_*X*_	*F*_*Y*_	*F*_*Z*_
7.9663	7.9359	8.0115	7.9653
